# Chemical Screening Method for the Rapid Identification of Microbial Sources of Marine Invertebrate-Associated Metabolites

**DOI:** 10.3390/md9030369

**Published:** 2011-03-21

**Authors:** Fabrice Berrue, Sydnor T. Withers, Brad Haltli, Jo Withers, Russell G. Kerr

**Affiliations:** Department of Chemistry & Department of Biomedical Sciences, Atlantic Veterinary College, University of Prince Edward Island, Charlottetown, PEI, Canada; E-Mails: fberrue@upei.ca (F.B.); sydwithers@gmail.com (S.T.W.); bhaltli@upei.ca (B.H.); jo.withers@yahoo.com (J.W.)

**Keywords:** chemical screening, coral-associated bacteria, UPLC/MS, gorgonian coral

## Abstract

Marine invertebrates have proven to be a rich source of secondary metabolites. The growing recognition that marine microorganisms associated with invertebrate hosts are involved in the biosynthesis of secondary metabolites offers new alternatives for the discovery and development of marine natural products. However, the discovery of microorganisms producing secondary metabolites previously attributed to an invertebrate host poses a significant challenge. This study describes an efficient chemical screening method utilizing a 96-well plate-based bacterial cultivation strategy to identify and isolate microbial producers of marine invertebrate-associated metabolites.

## 1. Introduction

Marine invertebrates are a well recognized source of new bioactive natural products with substantial potential for development as human therapeutics [1–7]. Despite the great potential of many natural products derived from marine invertebrates, the realization of this potential is frequently hindered by the inability of the natural source to provide sufficient quantities of the bioactive component to support traditional drug testing and development activities [8]. This “supply issue” is one of the greatest challenges facing the development of marine natural products.

There is a growing recognition that microorganisms associated with marine invertebrate hosts play an important role in the biosynthesis of secondary metabolites. As such, this could offer a new alternative to the production of potential drug-leads isolated from marine invertebrates [7,9,10]. Indeed, culturing invertebrate-associated microbes responsible for natural product biosynthesis would provide one approach to develop a sustainable supply of marine natural product leads [11]. However, the discovery of microorganisms producing secondary metabolites previously attributed to an invertebrate host has thus far, met with only modest success [12–14]. The isolation and cultivation of microbial symbionts remains as a significant challenge due to their potential dependence on host supplied factors for growth and secondary metabolite production [7,10].

This report describes a novel chemically oriented screen based on ultra high performance liquid chromatography/mass spectrometry (UPLC/MS) to direct the isolation of invertebrate-associated microorganisms implicated in the biosynthesis of secondary metabolites previously identified from the host invertebrate. This contrasts with the more conventional approach of prioritizing microbial isolates based on phylogenetic analysis and bioassay data. While this traditional approach is clearly of value, this provides only limited information on the ability of the isolates to produce novel secondary metabolites. A traditional chemical analysis of microbial culture extracts is of obvious value; however, this typically is a slow, tedious process. Herein, we describe a high-throughput chemically guided microbial isolation strategy which is facilitated by performing mixed bacterial cultures in a 96 multi-well plate (MWP) format and the use of new UPLC/MS technology as a rapid chemical screening tool. The high-throughput nature of the MWPs greatly facilitated the parallel investigation of multiple growth conditions while also greatly accelerating the chemical screening of MWP cultures [15]. The MWPs are employed for three separate culturing steps: (1) growth of mixed bacterial cultures derived from coral homogenate inocula in multiple media conditions, (2) growth of axenic cultures purified from mixed bacterial assemblages, and (3) optimization of media conditions to maximize yields of target compounds produced by bacteria purified from mixed cultures.

A chemical screening strategy can benefit from the use of standards (or target-molecules) which can be rapidly characterized by diagnostic parameters. Our approach utilizing UPLC/MS thus used retention time and MS data. Thus, the initial step of the study was a UPLC/MS investigation of the gorgonian coral *Erythropodium caribaeorum* (EC) in order to create an UPLC/MS library of target-molecules. EC was selected as a representative coral as it is abundant in many regions of the Caribbean and is known to possess a rich diversity of secondary metabolites [16–20]. MWP cultures of mixed microbial populations derived from coral homogenate inocula were performed in multiple growth conditions. Factors controlling secondary metabolite production in complex marine microorganism-invertebrate assemblages are largely unknown and thus we endeavored to expose the inoculated bacteria to a vast array of culture conditions which would hopefully allow for the establishment of an equally diverse number of cultures, thus increasing our chances of identifying a culture producing an EC metabolite. A 96 MWP format was utilized for the growth of axenic cultures purified from selected mixed microbial assemblages to identify the microbial producers of three secondary metabolites previously found in the gorgonian EC and secondly, to maximize production yield.

## 2. Results and Discussion

### 2.1. UPLC/MS Library of EC Secondary Metabolites

A UPLC/MS library of metabolites from EC was established to provide “standards” for the chemical screening of mixed bacterial cultures derived from EC tissue homogenates. Each member of the library was characterized by retention time, full MS and tandem MS/MS data of the most abundant ion using a standardized method. While the goal was not to identify each component of this mixture, amongst the 82 metabolites characterized in this manner, a few components could be readily identified by comparison with published data. The most abundant secondary metabolites present in EC extracts, erythrolides A and B (RT 2.05 min, 419.2 amu and RT 2.16 min, 479.1 amu, respectively) [16], were readily detected as were the potent antimitotic compounds, eleutherobin and desmethyleuleutherobin (RT 2.04 min, 657.2 amu, RT 1.69 min, 643.3 amu, respectively) [19].

### 2.2. Screening Mixed Cultures for “EC Metabolites”

The chemical diversity of EC is evident by the large number of metabolites readily detected by UPLC/MS. A mixed culture strategy employing a small scale 96 MWP growth format was used to query the relationship between the chemical diversity observed in EC extracts and the microbial community associated with this gorgonian. Employing this format, a homogenized EC tissue preparation was used to inoculate 1536 different culture conditions in order to expose the mixed microbial community to a variety of selective pressures and an array of nutritional resources with which to support secondary metabolite biosynthesis. We subsequently applied the UPLC/MS chemical screen previously used to create the EC metabolite library to analyze each of the 1536 mixed cultures for the production of the 82 metabolites initially observed in the coral extract. Direct chemical screening of such large numbers of samples has only recently been made feasible with the advent of UPLC technology which allows for the analysis of a large number of samples in a short period of time while increasing sensitivity and resolution [21–23]. For example, one 96 MWP plate can be analyzed by UPLC/MS and the data processed (using a customized application from the software Xcalibur^™^), allowing for the quantification of the 82 metabolites in each well in approximately 10 hours.

Four enrichment plates with 96 different conditions as described in Table 1 were prepared, and inoculated with EC homogenate and incubated at 30 °C and 37 °C under both static and shaken (150 rpm) conditions. The MWPs contained a nutritionally diverse array of media which varied in carbon source, nitrogen source and pH. The antibiotics penicillin G and kanamycin were added to select for the establishment of mixed cultures dominated by different groups of bacteria which exhibit natural resistance to these antibiotics. The goal of the enrichment plates was to generate a continuum of microbial diversity that could be further fermented and ultimately screened for the production of EC metabolites.

**Figure 1.**
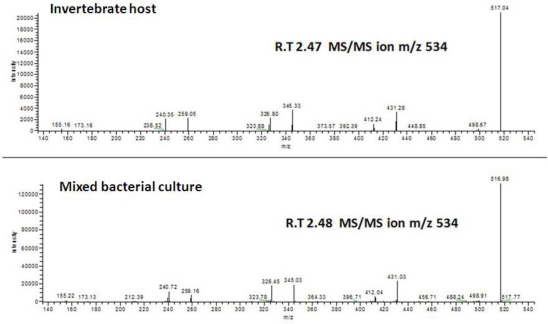
Comparison of the MS/MS spectra of extracts derived from the mixed bacterial culture and the coral *Erythropodium caribaeorum.*

After 7 days, the 4 enrichment plates representing 384 different cultures were subcultured into four 96-MWPs containing either the same array of media as the enrichment plates, or uniformly filled with Terrific Broth (TB), Marine Broth (MB) or 1/10 MB medium. Subcultured plates were incubated for an additional 14 days at 30 °C under static conditions to allow for the accumulation of secondary metabolites in the mixed cultures. Glycerol stocks were prepared from the four enrichment plates and the remaining culture broths (1.4 mL) were extracted and analyzed by UPLC/MS employing the same method used to generate the EC metabolite library. After 14 days of incubation, glycerol stocks from the 16 subcultured MWPs were prepared and the remaining culture broths were analyzed by UPLC/MS, and compared to the EC metabolite library. Among the 82 metabolites, three target molecules, compounds **41** (RT 2.48 min, 534.4 amu), **47** (RT 2.59 min, 620.3 amu) and, **53** (2.75 min, 706.3 amu) were detected in two mixed cultures across the 16 MWPs analyzed. The matches between the EC metabolite library and the bacterial mixed culture experiments were confirmed by comparison of their MS/MS spectra (Figure 1). Moreover, the MS data of compounds **41**, **47**, and **53** indicated that these three compounds belonged to a family of metabolites which possess varying amounts of repeating units with a mass of 86 amu.

### 2.3. Isolation and Identification of Pure Cultures Producing Target Compounds **41**, **47** and **53**

While it was interesting to confirm production of natural products **41**, **47** and **53** in mixed cultures, our goal was to use the described strategy to obtain pure cultures capable of producing compounds identified in the 82-member coral metabolite library. Thus, the MWP chemical screening strategy was used to guide the isolation of the microbial producer by testing growth cultures of single isolates. To obtain pure isolates of the bacterial producers of target compounds **41**, **47** and **53***,* glycerol stocks made from Subculture Plate 1 derived from Enrichment Plate 1 (plate 1–1) well D5 (TB medium + penicillin G) and Subculture Plate 1 derived from Enrichment Plate 2 (plate 2–1) well B1 (LB medium) were plated on TB agar and LB agar, respectively. Thirty six colonies were picked according their physical appearance and morphology and inoculated in 1.7 mL of NB, LB, TB, M9 glycerol and ASW glycerol media dispensed in a MWP. After 14 days at 30 °C, the plates were extracted and analyzed by UPLC/MS. Three colonies isolated from plate 1–1 well D5 (E1-1D5g, E1-1D5i, and E1-1D5j) and two colonies isolated from plate 2–1 well B1 (E2-1B1a and E2-1B1d) revealed the presence of the target compounds. Isolates E1-1D5j and E1-1D5g exhibited strong production in TB medium. The targeted compounds were also detected in the relatively nutritionally lean medium ASW glycerol for colonies E1-1D5i and E2-1B1a. The production of the target metabolites by these cultures was further confirmed by tandem mass spectrometry.

### 2.4. Structure Elucidation of Compounds **41**, **47** and **53**

The isolation and the identification of secondary metabolites from microorganisms often requires large culture volumes and multiple chromatographic steps in order to purify small amount of material from a complex mixture of microbial metabolites and media components. Our approach allowed us to rapidly identify and purify targeted compounds (**41**, **47**, and **53)** from the EC extract using standard chromatographic techniques. Compound **47** was characterized by 1D and 2D NMR analysis as well as tandem mass spectroscopy and identified as an oligomer containing seven 3-hydroxybutyric acid units. Due to observed differences of 86 amu in the mass spectra, compounds **41** and **53** were determined to possess six and eight 3-hydroxybutyric acid units, respectively.

### 2.5. Media Optimization

**Figure 2.**
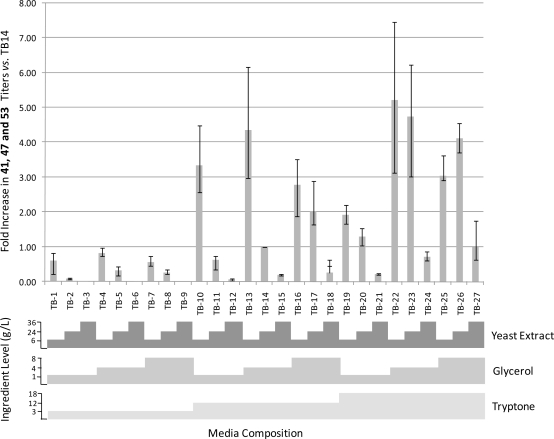
Results of Terrific Broth (TB) media optimization study.

To further validate the utility of the MWP cultivation format and to identify improved conditions for the production of **41**, **47** and **53** by EC isolates E1-1D5g, E1-1D5i, and E1-1D5j, a media study aimed at increasing metabolite yields was conducted. A full factorial approach to optimizing the TB media formulation was employed. Briefly, three arbitrary levels (low, medium and high) of the three primary nutritional media components (tryptone, yeast extract and glycerol) of TB medium were combined in all possible combinations, resulting in 27 media formulations which were dispensed in MWPs (Figure 2). Each of the 3 EC isolates were cultured in duplicate and the inoculated MWPs were incubated stationary for 14 days at 30 °C. Cultures were extracted and analyzed by UPLC/MS using methodology identical to that used to screen initial mixed cultures. The results of the media study are summarized in Figure 2. Several alternative TB medium formulations were identified which supported titers of **41**, **47** and **53** 1.3 to 5.23 fold greater than those supported by the standard TB medium formulation (TB14). Examination of Figure 2 indicates that yeast extract and tryptone concentrations had the most profound effect on formation of **41**, **47** and **53**. Increasing concentrations of yeast extract clearly inhibited their formation as titers consistently decreased in response to increasing yeast extract levels. Conversely, titers exhibited an increasing trend in response to increasing concentrations of tryptone. In general mid-level glycerol concentrations appeared to support greater production levels than the high or low concentrations, however, the effect of glycerol on the yield of **41, 47** and **53** was less pronounced than the effects of tryptone and yeast extract.

### 2.6. Taxonomic Identification of Bacteria Producing 3-Hydroxybutyric Acid Oligomers

To determine the taxonomic classification of the EC-derived colonies E1-1D5-g, -i, -j and E2-1B1-d, -a, the nearly complete 16S small subunit ribosomal RNA genes were amplified from 3 replicate colonies of each of the 5 isolates by PCR and the resulting amplicons were completely sequenced. Comparison of the 16S rDNA amplicons from the E1-1D5 isolates revealed that they were 100% identical, indicating that the three isolates were identical clones purified from well D5 of subculture plate 1–1. Comparison of the nearly full length 16S rRNA gene (1480 bp) to sequences contained within the GenBank non-redundant nucleotide database using the BlastN program indicated that the EC isolates shared 1479 out of 1480 residues with a number of *Photobacterium damselae* strains [24]. Comparison of the 16S rDNA amplicons from the E2-1B1-a and E2-1B1-d isolates also revealed that they were 100% identical, indicating that these isolates are identical clones purified from well B1 of subculture plate 2–1. Comparison of the nearly full length 16S rRNA gene (1547 bp) to sequences contained within the GenBank non-redundant nucleotide database using the BlastN program indicated that the 16S rDNA gene of EC isolates shared more than 98.5% identity with a number of *Vibrio harveyi* strains. The partial 16S rRNA gene sequences for *P. damselae* strain E1-1D5 and *V. harveyi* strain E2-1B1 have been deposited in GenBank under accession numbers HM143776 and HM143777, respectively.

## 3. Conclusions

A rapid MWP-based mixed culture strategy was successfully employed to screen more than 1500 small-scale mixed cultures for microbial producers of metabolites detected in organic extracts of the gorgonian coral EC. Small-scale MWP-based culturing paired with rapid UPLC/MS analysis affords an efficient method to not only identify natural products produced in mixed culture but also to evaluate axenic cultures. Inoculation of 1536 wells with EC tissue homogenate and subsequent rapid chemical screening by UPLC/MS resulted in the detection of 2 wells containing elevated quantities of three related metabolites identified in the EC extract. The three metabolites were revealed to be 3-hydroxybutyric acid oligomers containing 6 to 8 monomers by interpretation of NMR and LC/MS data. These compounds have not previously been reported from EC. Two axenic strains of bacteria were isolated from glycerol stocks prepared from the two wells originally exhibiting production, and were subsequently shown to be strains of *P. damselae* and *V. harveyi* via 16S rRNA gene sequencing. Both *Photobacterium* and *Vibrio* are known to produce polyhydoxybutyrate (PHB) oligomers and polymers and other polyhydroxyalkanoates [25–27]. In bacteria, PHB is commonly encountered as a high molecular weight polymer (150–30,000 monomer units) and is usually accumulated in intracellular light refractive granules when an essential nutrient such as nitrogen or phosphorous is limited in the presence of excess carbon source, and is believed to function as an energy reserve [28,29]. *Photobacterium* and *Vibrio* are both ubiquitous marine bacteria belonging to the *Vibronacea* which are often associated with disease states in a number of marine organisms. The role of these bacteria in EC is not clear at this time; however, these microbes were isolated from tissue exhibiting no outward sign of disease.

The approach described above to identifying microbial producers of metabolites present in marine invertebrates represents a unique and rapid method which should be applicable to a variety of systems. Traditional strategies have relied on the isolation of axenic cultures from the invertebrate followed by growth under multiple culture conditions in hopes of identifying a microbial producer of the targeted metabolite. The latter approach suffers from a number of drawbacks. Firstly, significant resources are expended isolating, culturing and analyzing microbes which do not possess the capability to produce the compounds of interest. Secondly, culturing of axenic isolates may not provide the biochemical signals normally present in a complex microbial community necessary for the induction of secondary metabolite biosynthesis. Bacteria are well known to employ a number of biochemical signals to control gene expression, and natural product biosynthesis under laboratory conditions has been shown to rely on signaling between different species of a marine bacterial population [30,31]. Screening mixed cultures for metabolite production avoids these potential pitfalls while simultaneously allowing for the subsequent purification of axenic cultures from archived frozen glycerol stocks. The described methodology was also validated as a rapid and cost-effective tool for the preliminary optimization of growth conditions prior to more costly large scale cultures.

## 4. Experimental Section

### 4.1. Identification of Secondary Metabolites Present in EC

Samples of EC (5 kg) were collected off the coast of Florida at a depth of 10 m in June 2007 and were extracted several times with a mixture ACN:DCM (1:1). The crude extract was fractionated by flash chromatography on C18 and the resulting fractions were further purified by normal phase Diol with a step gradient hexane-EtOAc-MeOH and reversed phase C18 column chromatography with a H_2_O-MeOH-DCM stepwise gradient system. All the fractions were analyzed by UPLC (Thermo Scientific, Accela) coupled to an ELSD (Sedere, Sedex LT-ELSD Model 80LT) and mass spectrometer (Thermo Scientific, LTQ ion trap) with a standard gradient from 5% ACN in H_2_O (0.1% formic acid in both eluents) to 100% ACN in 6 minutes.

### 4.2. Chemical Screening of MWP Mixed Cultures

*E. caribaeorum* was collected at Boca Raton, Florida in February 2007 by SCUBA at a depth of 13 m. Coral specimens were individually flash frozen with liquid nitrogen and subsequently stored at −80 °C. Flash-frozen coral (1 g) was homogenized in 50 mL artificial sea water (ASW) using a commercial Waring blender. The contents were filtered through four layers of sterile cheesecloth and 17.5 μL of coral homogenate was used to inoculate each well of 4 enrichment plates (96 MWPs) containing 1.7 mL of culture broth per well. The enrichment plates were composed of gradients of pH, salt, nitrogen source, carbon source and antibiotics (penicillin G or kanamycin) as illustrated in the Table 1. All media were prepared with deionized water and autoclaved to sterilize the medium unless otherwise noted. Media compositions were as follows: Emerson medium—yeast extract (10.0 g/L), beef extract (44 g/L), peptone (4 g/L), NaCl (25 g/L), glucose (10 g/L, added post sterilization) [32]; Artificial Sea Water (ASW) medium—Instant Ocean™ (36 g/L) and glucose or glycerol (4 g/L, added post sterilization); R2 medium—yeast extract (0.5 g/L), proteose peptone (0.5 g/L), casamino acids (0.5 g/L), soluble starch (0.5 g/L), sodium pyruvate (0.3 g/L), K_2_HPO_4_ (0.3 g/L), MgSO_4_ (0.05 g/L), glucose (0.5 g/L, added post sterilization), pH 7.2 [33]; ISP1 medium—pancreatic digest of casein (5 g/L), yeast extract (3 g/L), nalidixic acid (10 μg/mL post sterilization) [34]; M9 media—Difco™M9 Minimal Salts 5X powder (11.28 g/L) and glycerol or glucose or arabinose (4 g/L, added post sterilization) [35]; MB—Difco™ Marine Broth 2216 (37.4 g/L) [36]; 1/10MB—Difco™ Marine Broth 2216 (3.74 g/L), NaCl (18 g/L); NB—EMD Nutrient Broth powder (8 g/L) [37]; 1/10NB—EMD Nutrient Broth powder (0.8 g/L); NB/SW—EMD Nutrient Broth powder (8 g/L), Instant Ocean™ (36 g/L); NB 0.5% butanol—EMD Nutrient Broth powder (8 g/L), butanol (5 mL/L); LB—EMD LB Broth Miller powder (25 g/L); TB—EMD TB powder (47.6 g/L) [35]. Buffered NB, 1/10 NB, MB and 1/10 MB were generated by preparing the media as described above with the addition of the following 0.1 M Na_2_HPO_4_·7H_2_O and 0.1 M NaH_2_PO_4_·H_2_O per litre of media; pH 5.8, 7.9 mL 0.1 M Na_2_HPO_4_·7H_2_O and 92.1 mL 0.1 M NaH_2_PO_4_·H_2_O; pH 6.5, 30.3 mL 0.1 M Na_2_HPO_4_·7H_2_O and 69.7 mL 0.1 M NaH_2_PO_4_·H_2_O; pH 7.2, 68.4 mL 0.1 M Na_2_HPO_4_·7H_2_O and 3.16 mL 0.1 M NaH_2_PO_4_·H_2_O; pH 8.0, 93.2 mL 0.1 M Na_2_HPO_4_·7H_2_O and 6.8 mL 0.1 M NaH_2_PO_4_·H_2_O. Penicillin G and kanamycin were added to wells as at a concentration of 10 μg/mL and 50 μg/mL, respectively. Following inoculation, the four enrichment plates were covered with a sterile breathable nylon adhesive membrane and incubated for 7 days under the following conditions: Enrichment Plate 1, 30 °C/150 rpm; Enrichment Plate 2, 37 °C/150 rpm; Enrichment Plate 3, 30 °C/static; Enrichment Plate 4, 37 °C/static. Each enrichment plate was then subcultured using a 96-pin replicator (Boekel Scientific, USA) in four different 96 MWPs (Table 1). The first plate possessed the same layout as the enrichment plate (subculture plate 1) and the three other plates were uniformly filled with TB (Subculture Plate 2), MB (Subculture Plate 3) or 1/10MB (Subculture Plate 4). The sixteen resulting subculture plates were incubated static at 30 °C for 14 days. Prior to harvesting, each plate was archived by transferring 100 μL from each well to shallow 96 MWPs and mixed with 100 μL 50% glycerol (w/v) and stored at −80 °C. The plates were centrifuged at 3000 × *g* at 24 °C for 20 min, and 1 mL of supernatant was transferred to a new 96 MWP and dried under vacuum using a Genevac EZ-2 instrument (Genevac Inc, USA). Sterile deionized H_2_O (100 μL) was added to the dried residues and incubated for 10 min at room temperature with occasional shaking. ACN (900 μL) was added to the rehydrated residues and mixed thoroughly by repeated aspiration through a 1 mL pipette tip. The resulting extract was centrifuged at 1000 × *g* for 5 min. The supernatant was transferred to a new plate, dried under vacuum and dissolved in 100 μL of MeOH. Extracts were analyzed by UPLC/MS using the same method used to generate the EC target-molecule library.

### 4.3. Chemical Screening of Axenic MWP Cultures

A portion (5–10 μL) of mixed culture glycerol stock of cultures which showed production of metabolites identified in EC extracts were diluted 50 fold in TB and LB liquid media and plated on TB agar (media prepared as for liquid media described above but amended with 15 g/L agar) and incubated at 30 °C for 7 days. Two representative colonies of each morphological type were used to inoculate the wells of a new MWP containing 1.7 mL of the following media: NB, LB, TB, M9 glycerol and ASW glycerol. Plates were incubated static at 30 °C for 14 days. Plates were extracted and analyzed in an identical fashion to that used for the original mixed culture plates.

### 4.4. TB Media Optimization Study

The standard TB medium formulation consists of tryptone (12 g/L), yeast extract (24 g/L), glycerol (4 g/L), K_2_HPO_4_ (9.4 g/L) and KH_2_PO_4_ (2.2 g/L). All media formulations contained identical concentrations of K_2_HPO_4_ and KH_2_PO_4_ while the levels of tryptone, yeast extract and glycerol were varied between low, mid and high levels (trypone: low 3 g/L, mid 12 g/L, high 18 g/L; yeast extract: low 6 g/L, mid 24 g/L, high 36 g/L; glycerol: low 1 g/L, mid 4 g/L, high 8 g/L). All media were autoclaved at 121 °C for 15 min and 1.5 mL was dispensed in the wells of sterile MWPs. To prepare inocula for MWP cultures, three individual isolates (E1-1D5-g, -i, and –j) were used to inoculate 10 mL of TB (standard formulation) medium and cultured for 16 hr at 30 °C and 150 rpm. A portion (5 μL) of these cultures were used to inoculate each well of the MWP. Duplicate MWP cultures were incubated at 30 °C under static conditions for 14 days and were extracted and analyzed as described above for mixed microbial cultures.

### 4.5. Structure Determination

EC crude extract (100g) was partitioned between MeOH and hexane. After evaporation under vacuum, the MeOH extract (42 g) was consecutively fractionated by flash chromatography on C18 (Phenomenex, Sepra *C18*-T (50 μm, 135 Å)) with a step gradient H_2_O:MeOH (9:1), H_2_O:MeOH (5:5), H_2_O:MeOH (2:8), ACN, acetone and DCM:MeOH (1:1) and flash chromatography on Diol with a step gradient of hexane to EtOAc to MeOH. The fraction (188 mg) containing the target compounds were further fractionated using an automated C18 chromatography system (Teledyne, Combiflash Rf) and compound **47** was finally purified by reversed phase HPLC (Phenomenex luna C18 5u, 250 × 10 mm) using an isocratic gradient H_2_O [0.1% FA]:ACN [0.1% FA] (30:70) to yield to a white powder (2.9 mg). Compound **47** was characterized by 1D and 2D NMR analysis (Bruker Avance 600 MHz) as well as tandem mass spectroscopy and identified as an oligomer containing seven 3-hydroxybutyric acid units. ^1^H NMR (300 MHz, CDCl_3_) δ 5.30 (1H, m), 2.63 (1H, dd, *J* = 7.7, 15.5 Hz), 2.48 (1H, dd, *J* = 5.4, 15.5 Hz), 1.29 (3H, d, *J* = 6.4 Hz); ^13^C NMR (75 MHz, CDCl_3_) δ 169.4 (s), 67.8 (d), 40.9 (d), 19.9 (q). Due to observed differences of 86 amu in the mass spectra, compounds **41** and **53** were determined to possess six and eight 3-hydroxybutyric acid units, respectively.

### 4.6. 16S rDNA Sequencing

16S ribosomal RNA gene fragments were amplified using the GoTaq Green Master Mix PCR kit (Promega) according to the manufacturer’s specifications. Reactions (50 μL) contained 0.5 μM of each of the universal bacterial 16S rRNA primers E8F (5′-AGAGTTGATCCTGGCTCAG) and E1541R (5′-AAGGAGGTGATCCANCCRCA) [38]. Template DNA was prepared by dispersing actively growing single colonies picked from TB agar plates in 50 μL of PCR grade DMSO. A portion of the DMSO cell suspension (1.25 μL) was added to each PCR reaction resulting in a final DMSO concentration of 5%. Thermal cycling parameters consisted of an initial denaturing step of 3 min at 94 °C and 30 cycles consisting of 1 min at 94 °C, 1 min at 54 °C and 2 min at 72 °C and a final extension step at 72 °C for 10 min. PCR products were purified using the DNA Clean and Concentrator-5 kit (Zymo Research Corporation). Purity and concentration of purified PCR products were assessed by agarose gel electrophoresis. PCR products were sequenced by The Centre for Applied Genomics (Toronto, CA).

## Figures and Tables

**Table 1.** Enrichment plate layout.

	**No Antibiotics**				**+Penicillin G**				**+Kanamycin**			
	**1**	**2**	**3**	**4**	**5**	**6**	**7**	**8**	**9**	**10**	**11**	**12**
**A**	NB	NB pH 5.8	1/10 NB pH 5.8	MB pH 5.8	NB	NB pH 5.8	1/10 NB pH 5.8	MB pH 5.8	NB	NB pH 5.8	1/10 NB pH 5.8	MB pH 5.8
**B**	LB	NB pH6.5	1/10 NB pH6.5	MB pH6.5	LB	NB pH6.5	1/10 NB pH6.5	MB pH6.5	LB	NB pH6.5	1/10 NB pH6.5	MB pH6.5
**C**	MB	NB pH 7.2	1/10 NB pH 7.2	MB pH 7.2	MB	NB pH 7.2	1/10 NB pH 7.2	MB pH 7.2	MB	NB pH 7.2	1/10 NB pH 7.2	MB pH 7.2
**D**	TB	NB pH 8.0	1/10 NB pH 8.0	MB pH 8.0	TB	NB pH 8.0	1/10 NB pH 8.0	MB pH 8.0	TB	NB pH 8.0	1/10 NB pH 8.0	MB pH 8.0
**E**	dH_2_0	ISP1 10μg/mL NA	NB + 0.5% butanol	1/10 MB pH 5.8	dH_2_0	ISP1 10μg/mL NA	NB + 0.5% butanol	1/10 MB pH 5.8	dH_2_0	ISP1 10μg/mL NA	NB + 0.5% butanol	1/10 MB pH 5.8
**F**	M9 glycerol	NB/SW	ASW	1/10 MB pH6.5	M9 glycerol	NB/SW	ASW	1/10 MB pH6.5	M9 glycerol	NB/SW	ASW	1/10 MB pH6.5
**G**	M9 glucose	Emerson	ASW glycerol	1/10 MB pH 7.2	M9 glucose	Emerson	ASW glycerol	1/10 MB pH 7.2	M9 glucose	Emerson	ASW glycerol	1/10 MB pH 7.2
**H**	M9 arabinose	R2 Broth	ASW glucose	1/10 MB pH 8.0	M9 arabinose	R2 Broth	ASW glucose	1/10 MB pH 8.0	M9 arabinose	R2 Broth	ASW glucose	1/10 MB pH 8.0
